# Ticking time bombs: connections between circadian clocks and cancer

**DOI:** 10.12688/f1000research.11770.1

**Published:** 2017-10-30

**Authors:** Katja A. Lamia

**Affiliations:** 1 Department of Molecular Medicine, The Scripps Research Institute, 10550 North Torrey Pines Road, La Jolla, CA, 92037, USA

**Keywords:** circadian clock, cancer, cell cycle, PERs, CRYs

## Abstract

Connections between mammalian circadian and cell division cycles have been postulated since the early 20th century, and epidemiological and genetic studies have linked disruption of circadian clock function to increased risk of several types of cancer. In the past decade, it has become clear that circadian clock components influence cell growth and transformation in a cell-autonomous manner. Furthermore, several molecular mechanistic connections have been described in which clock proteins participate in sensing DNA damage, modulating DNA repair, and influencing the ubiquitination and degradation of key players in oncogenesis (c-MYC) and tumor suppression (p53).

## Introduction

Connections between mammalian cell division cycles and time of day have been postulated since the early 20th century when Mrs. C.E. Droogleever Fortuyn-van Leijden demonstrated that the difficulty of observing mitosis in growing tissues stemmed from its propensity to occur late at night ^1^. Similar daytime-dependent changes in the mitotic indices of several rodent tissues were reported by 1950 ^2, 3^. By the 1960s, it became clear that many biological daily rhythms are driven by endogenous oscillators, and the term “circadian” was adopted to describe endogenous rhythms with a period close to that of the 24-hour day ^4^. Halberg and Barnum demonstrated the existence of circadian rhythms in DNA synthesis and mitosis in healthy mouse tissues *in vivo* ^5^. Circadian rhythms of cell division in human proliferating cell populations *in vivo* have also been documented ^6, 7^. Careful examinations of the relationship between circadian and cell division cycles in individual proliferating fibroblasts in cell culture have demonstrated that cell division is influenced by circadian time but is not limited to a specific circadian phase ^8– 10^, suggesting a complex relationship between these two biological oscillators. Many epidemiological studies have demonstrated that disruption of circadian rhythms caused by shift work increases the risk of several cancers ^11– 18^, and the size of the effect is correlated with the duration and severity of circadian disruption. Thus, long-term rotating shift work confers the greatest increase in risk. Notably, unlike other tumor types, skin cancers were recently found to be reduced among night shift workers ^19^ and this might be due to reduced sun exposure. Accumulated evidence for increased risk of several cancers in shift workers led the World Health Organization to declare circadian disruption a probable carcinogen ^14^. However, controversy remains over the generality and robustness of these effects ^20– 23^, and some have raised concerns that lifestyle factors associated with shift work may enhance cancer risk independent of disruption of circadian rhythms per se. Conversely, several studies have found significant effects of genetic variants or expression level of clock genes on human cancer incidence or survival ^24, 25^ or on the tumor burden in genetically engineered mouse models of cancer ^26, 27^. While circadian rhythms clearly influence cell division and tumor formation, we are only beginning to understand the molecular underpinnings for their interrelationship.

Mammalian circadian clocks are most widely recognized as the drivers of sleep cycles. Such behavioral rhythms are driven by secreted factors from the suprachiasmatic nucleus (SCN), a neuronal master pacemaker located at the base of the anterior hypothalamus, just above the optic chiasm ^28, 29^. Konopka and Benzer’s elucidation of the genetic basis for circadian activity rhythms in fruit flies provided the first evidence for genetically determined behavior ^30^ and jump-started research in eukaryotic molecular chronobiology. Subsequent work has demonstrated that mammalian circadian behavior is also genetically determined ^31^ and defined a transcription-translation feedback loop that drives cell-autonomous rhythms of gene expression in nearly all mammalian cells ^32^. The core molecular clock is driven by a heterodimer of the basic helix-loop-helix transcription factor BMAL1 with either CLOCK or NPAS2, which activates the expression of thousands of genes, including those encoding period (PER1-3) and cryptochrome (CRY1,2) proteins, which repress CLOCK/BMAL1 activity, and the nuclear hormone receptors REV-ERBα and REV-ERBβ, which repress *Bmal1* expression. TIMELESS is the mammalian homolog of *Drosophila melanogaster* TIM (dTIM), which dimerizes with dPER and is required for circadian rhythms in flies. The mechanistic role of TIMELESS in mammalian clocks is unclear, but it is required for maintenance of normal circadian rhythms ^33, 34^.

The state of our understanding of the connections between circadian rhythms and cell division today is reminiscent of the early days investigating connections between clocks and metabolism, when there was considerable resistance to the idea that circadian rhythms could modulate metabolic function at the molecular level. Only after it was established that circadian rhythms in individual organs modulate metabolic physiology independent of behavioral and feeding rhythms ^35– 37^ has it become possible to dissect specific mechanisms by which clocks regulate metabolic pathways in a cell- and tissue-autonomous manner. The past decade has seen several important advances in understanding molecular connections between core components of molecular circadian clocks and cell division, including some of the most frequently mutated players in human cancer. Our understanding of the role of clocks in cancer development is still in its infancy and will greatly benefit from enhanced communication, interaction, and resource sharing among experts in circadian rhythms, cell division, and cancer biology.

## Tumor studies in mice

Several studies in animal models support the hypothesis that circadian clocks control cell proliferation or transformation (or both) independent of other lifestyle changes ( Table 1). Early studies found that the timing of cell division after partial hepatectomy in rats displays a robust circadian rhythm antiphase to the rhythmic production of endogenous corticosteroids ^38^. Later, Okamura and colleagues reproduced those findings in mice and showed that genetic disruption of circadian clock components altered the timing of the first cell division ^39^. Lévi and colleagues demonstrated that surgical ablation of the SCN or “master clock” greatly enhanced the growth of implanted tumors in addition to abolishing circadian rhythms of behavior and body temperature ^40^. Like the difficulty in separating direct cell-autonomous clock control of metabolic functions from effects on behavior (feeding/activity cycles), these studies cannot distinguish between effects of systemic circadian control of daily fluctuations in feeding, hormone production, and so on that may indirectly influence cell growth and division. Indeed, it seems likely that the effects of circadian disruption on cancer risk are multi-faceted and could involve both cell-autonomous and systemic effects.

**Table 1.** Effects of genetic and environmental circadian disruption in mouse cancer models.

Disruption	Location	Impact	Reference(s)
*Bmal1 ^−/−^*	Ubiquitous	Enhanced *Kras ^G12D^* lung tumors	26
*Bmal1 ^−/−^*	Hepatocytes	Enhanced hepatocellular carcinoma (HCC) and prevented further increase in response to chronic jet lag	41
*Bmal1 ^−/−^*	Lung epithelium	Enhanced *Kras ^G12D^* and *p53 ^−/−^;Kras ^G12D^* lung tumors	26
*Bmal1 ^−/−^*	Keratinocytes	Reduced RAS-driven squamous tumors	53
*Cry2 ^−/−^*	Ubiquitous	Enhanced lymphoma in *Emu-MYC*	27
*Cry1 ^−/−^;Cry2 ^−/−^*	Ubiquitous	Decreased tumor formation in *p53* ^−/−^; enhanced HCC and cholangiocarcinoma	41, 42, 46, 47
*Per2 ^m/m^*	Ubiquitous	Enhanced tumors caused by irradiation, diethylnitrosamine, or mutant *Kras* or *p53*	26, 43, 45
*Per2 ^S662G^ or* *Per2 ^S662D^*	Ubiquitous	Enhanced tumor formation in *p53 ^R172H^* mice	44
*Per1 ^−/−^;Per2 ^−/−^*	Ubiquitous	Enhanced HCC	41
Chronic jet lag	Environmental	Enhanced tumor formation in breast, lung, and liver models	26, 41, 54– 56

Several studies have examined the effect of ubiquitous deletion or mutation of the circadian repressors *Cry1/2* and *Per1/2* on tumor incidence. Deletion or mutation of *Per2* either alone or in combination with deletion of *Per1* has consistently been found to increase the incidence of tumor formation in several different genetic or irradiation-induced tumor models ^26, 41– 45^. Reported effects of *Cry1* or *Cry2* deletion (or both) on tumor formation have varied. While deletion of both *Cry1* and *Cry2* improves survival and decreases the tumor burden in *p53* ^−/−^ mice ^46^, the same double deletion enhances spontaneous ^41^ and irradiation-induced ^42^ formation of hepatocellular carcinomas (HCCs) and increases the formation of cholangiocarcinomas after exposure to diethylnitrosamine ^47^. These differences may be due to unique functions of CRY1 and CRY2 ^27, 48^ and differences in the molecular pathways targeted in each tumor model. Consistent with this hypothesis, deletion of *Cry2* alone consistently enhances cellular transformation in cooperation with multiple different oncogenic manipulations, whereas deletion of *Cry1* decreases transformation only in the context of *p53* depletion ^27^. Furthermore, loss of *Cry2* increases the formation of MYC-driven lymphomas in mice with wild-type *Cry1* ^27^. Additional studies of CRY1 and CRY2 are needed to understand their overlapping and distinct roles in cell division and tumor formation. New genetic tools for tissue-specific ablation of *Cry1/2* and *Per1/2/3* will enable the elucidation of their effects on cell-autonomous growth and survival and global physiology. Additional studies investigating the effects of clock gene disruptions in tumor models driven by a variety of genetic manipulations (and in myriad cell types) are also needed to improve our understanding of how circadian disruption impacts different types of cancers.

Recently, tissue-specific ablation of clock function via Cre-mediated deletion of *Bmal1* in lung epithelial cells, in conjunction with other genetic manipulations to induce local tumor formation, demonstrated that loss of the tumor-resident circadian clock enhanced lung tumor progression ^26^. The hypothesis that BMAL1 opposes cell proliferation in a cell-autonomous manner is supported by studies of normal and transformed rodent cell lines ^49^, N-MYC driven glioblastoma cell lines ^50^, and deletion of *Bmal1* in keratinocytes *in vivo* ^51^. Perhaps not surprisingly, many transformed cell lines exhibit altered or lost circadian rhythms ^52^; restoration of clock function in B16 melanoma cells reduced proliferation both in culture and after implantation in mice ^53^. However, another study found that keratinocyte-specific *Bmal1* deletion reduced the incidence of RAS-driven squamous tumors ^54^. Thus, the effect of *Bmal1* deletion on cell growth and transformation may depend on the cellular or genetic context in which it occurs.

A handful of recent studies demonstrated that exposing mice to light cycles engineered to impose a state of “chronic jet lag”, mimicking the experience of rotational shift work, increased tumor formation in breast, lung, and liver cancer models ^26, 41, 55– 57^. Liver-specific deletion of *Bmal1* prevented the increase in HCC caused by chronic jet lag, suggesting a tumor-autonomous effect of circadian disruption ^41^. It will be interesting to further investigate how specific genetic manipulation of clock components alters the impact of light cycle changes to determine the primary molecular mechanism(s) by which circadian disruption impacts tumor initiation or progression or both.

## Emerging molecular connections

**Figure 1.**
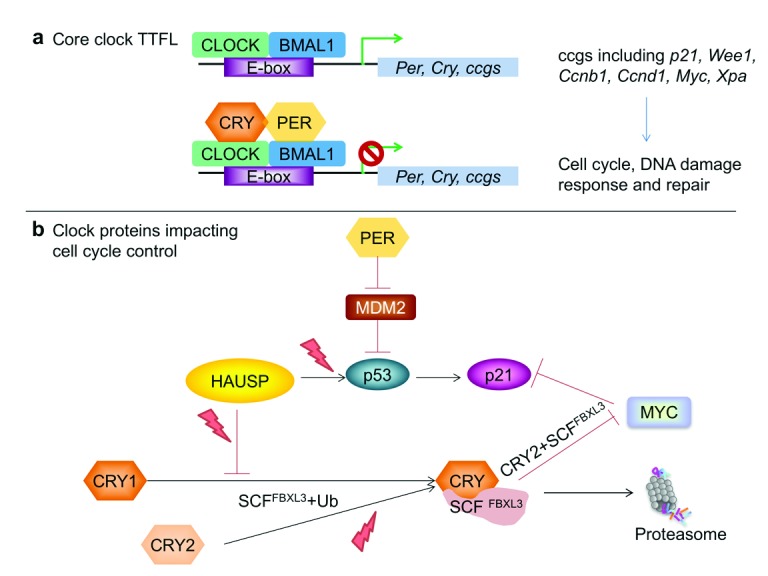
Molecular connections between circadian clocks, cell cycle, and cancer drivers. ( **a**) The core mammalian circadian clock transcription-translation feedback loop (TTFL) involves the positive factors CLOCK and BMAL1 activating expression of their own repressors PERs and CRYs. This clock mechanism also drives daily rhythmic expression of so-called clock-controlled genes (ccgs), including *P21* ( *Cdkn1a*), *Wee1*, *Ccnb1*, *Ccnd1*, *Myc*, and *Xpa* mRNAs. ( **b**) PER and CRY modulate post-translational regulation of P53 and c-MYC. PER2 blocks MDM2 ubiquitination of P53, while CRY2 stimulates ubiquitination of c-MYC by SCF(FBXL3). HAUSP removes polyubiquitin chains from CRY1 as well as from P53. Lightning bolts represent processes that are stimulated by DNA damage. Additional connections are described in the text.

Several studies have demonstrated a non-random association between the timing of the circadian cycle and that of the cell cycle ^8– 10^. Although the relationship between these two oscillators is not well understood, some molecular connections have been described ( Figure 1), including circadian transcriptional regulation of the key cell cycle regulators *Wee1*, *p21*, *Ccnb1*, and *Ccnd1* (encoding CYCLINs B1 and D1) ^39, 58– 60^. *Wee1* transcription can be directly activated by CLOCK/BMAL1 and repressed by PERs or CRYs ^39^. PER1 influences the transcription of *Wee1* and *Ccnb1* by a p53-dependent mechanism and of *p21* independent of p53, possibly by stabilizing c-MYC ^58^. Circadian clocks may also influence cell cycle regulators indirectly by modulating the activity of critical signal transduction cascades that alter cell cycle dynamics. A genome-wide screen for modulators of circadian rhythm found an overrepresentation of phosphatidylinositol 3-kinase effectors ^61^, which is also a key pathway for modulating cell cycle and cell proliferation ^62^. *In vivo*, endogenous glucocorticoids exhibit high-amplitude circadian rhythms and inhibit signaling downstream of the epidermal growth factor receptor (EGFR) via glucocorticoid receptor-induced activation of EGFR pathway inhibitors ^63^.

### Clock input to DNA damage response and repair

Consistent with observed rhythms in mitotic indices, several studies have demonstrated circadian rhythms of sensitivity to various types of DNA damage. Mouse skin and hair follicles exhibit maximum sensitivity to DNA damage at night induced by either ultraviolet (UV) or ionizing radiation ^51, 64^. Rhythms in sensitivity to damage were lost in mice harboring genetic deletion of *Bmal1* in keratinocytes or ubiquitous deletion of *Cry1* and *Cry2*. Interestingly, both (6-4) photoproducts (64Ps) and cyclobutane pyrimidine dimers (CPDs) are reduced, but double-strand breaks (DSBs) are increased, across the circadian cycle in *Bmal1*-deficient skin ^51^. 64Ps and CPDs can be removed by nucleotide excision repair (NER), which exhibits circadian rhythms in mouse brain and liver lysates ^65, 66^. Rhythmic NER is likely due to circadian rhythms in mRNA and protein expression of xeroderma pigmentosum complementation group A (XPA), a zinc finger nuclease that directly recognizes and repairs photoproducts and DNA adducts induced by chemical carcinogens ^65^. Elevated XPA could contribute to reduced 64Ps and CPDs in *Bmal1*-deficient skin exposed to radiation without affecting the incidence of DSBs. In addition to demonstrating rhythms in sensitivity to damage, several studies have documented circadian rhythms in intracellular concentrations of reactive oxygen species (ROS) ^51, 67, 68^, which can be a source of genome insult. Those rhythms may have provided evolutionary impetus that favored connections between circadian clocks and DNA damage response and repair pathways. Oscillations in intracellular ROS may result from circadian control of cellular metabolism and may be related to recently described oscillations in cell and tissue oxygenation and hypoxia-responsive signaling ^69– 71^.

CRY1 and CRY2 evolved from bacterial UV-activated DNA repair enzymes ^72^, and several studies suggest that they retain a functional role in genome protection. Although they lack catalytic DNA repair activity, purified human CRY2 retains the ability to preferentially interact with single-stranded DNA containing a UV photoproduct *in vitro* ^73^. Furthermore, CRY2-deficient cells exhibit increased accumulation of DNA DSBs ^24, 48^. CRY1 and CRY2 are phosphorylated on unique sites following DNA damage, resulting in stabilization of CRY1 and degradation of CRY2 ^48, 74^. Furthermore, they play overlapping and distinct roles in modulating the transcriptional response to DNA damage ^48^. While some of the transcriptional changes in *Cry2* ^−/−^ cells can be explained by the unique role of CRY2 in modulating c-MYC protein stability (see below), further investigation will be required to understand the mechanism(s) by which mammalian CRYs participate in the DNA damage response.

Although the precise role of TIMELESS in mammalian circadian clocks is not well defined, it clearly impacts clock function in mammals ^33, 34^ and interacts with mammalian CRY1 ^34^ and CRY2 ^75^. It also directly interacts with PARP-1 and thereby is recruited to sites of DNA damage ^76^. Depletion of TIMELESS or replacement with a mutant that cannot interact with PARP-1 greatly reduced homologous recombination repair ^76^. These recent findings likely explain earlier observations that depletion of TIMELESS reduced the activation of checkpoint kinases 1 (CHK1) and 2 (CHK2) in response to DNA damage ^75, 77, 78^. CLOCK is also recruited to DNA DSBs independent of H2AX ^79^, although no functional impact of CLOCK deficiency on the DNA damage response has been established.

### Regulation of protein turnover of key cancer drivers

Several recent studies have uncovered unexpected roles for CRY1, CRY2, and PER2 in modulating the targeting of substrates for ubiquitination, including two of the most commonly mutated proteins in human cancers: p53 and c-MYC. PER2 interacts directly with p53 and prevents its ubiquitination by the MDM2 E3 ubiquitin ligase, resulting in stabilization of p53 in cells expressing high levels of PER2 ^80, 81^. This may explain earlier observations that thymocytes from *Per2* mutant mice are deficient in p53 stabilization after irradiation ^43^. In addition, PER2 seems to modulate p53 nuclear import ^82^, perhaps via effects on p53 ubiquitination. The herpes virus-associated ubiquitin-specific protease (HAUSP) removes polyubiquitin chains from both MDM2 and p53 ^83– 87^. Its affinity for MDM2 is reduced and for p53 is increased following DNA damage, contributing to stabilization of p53. HAUSP also interacts with CRY1 through its C-terminal tail, which is not conserved in CRY2, and this interaction is increased in response to DNA damage, resulting in stabilization of CRY1 while CRY2 is destabilized ^48^.

In response to DNA damage, the interaction between CRY2 and the E3 ligase substrate adaptor F-box and leucine-rich repeat 3 (FBXL3) is increased ^48^. FBXL3 targets both CRY1 and CRY2 for ubiquitination by a SKP-CULLIN-Fbox (SCF) E3 ligase complex ^88^, and mutation of FBXL3 alters circadian period length ^89, 90^. In addition to being substrates of FBXL3-mediated ubiquitination, CRY1 and CRY2 influence the formation of FBXL3-containing SCF complexes ^91^ and CRY2 recruits phosphorylated c-MYC to SCF(FBXL3) ^27^. Indeed, disruption of CRY2 or FBLX3 stabilizes c-MYC as much as depletion of its best established E3 ligase FBXW7 ^27^. Consistent with this, c-MYC was increased in lung tumors subject to genetic disruption of clock function ^26^. Furthermore, c-MYC protein exhibits circadian oscillation in mouse thymus and is elevated throughout the day upon exposure to chronic jet lag ^42^. CRY1 and CRY2 may also stimulate the ubiquitination of other substrates by SCF(FBXL3) or other E3 ligases. In fruit flies, dCRY is required for ubiquitination of dTIM by JETLAG in response to blue light ^92^, and mammalian CRY1 was recently found to be involved in MDM2-mediated ubiquitination of FOXO1 in mouse livers ^93^. PER1 has also been shown to alter the protein stability of both p53 and c-MYC ^58^; it is unclear whether these effects are indirectly caused by altered expression of PER2 or CRY2 or both. In addition, PER1 and PER2 have been reported to interact with the RNA binding protein NONO and thereby contribute to circadian activation of *p16Ink4A* expression ^94^. Thus, inactivation of PERs could inhibit both the retinoblastoma (Rb) and p53 tumor suppressors.

## Looking ahead

Several studies have found that circadian rhythms tend to be reduced or absent in tumors, that this can be driven by acute induction of individual oncogenes ^50, 52^, and even that tumors can dampen circadian rhythms in remote organs ^95^. Patients with cancer often experience disruption of sleep-wake cycles and other systemic circadian rhythms, and those disruptions are associated with poor outcomes ^96^. Interventions to improve the robustness of overall circadian timing systems in these patients may be beneficial.

Circadian disruption in shift workers enhances the risk of several types of cancer. Molecular connections between mammalian clock components and critical regulators of cell proliferation and survival suggest several possible underlying mechanisms that could explain those phenomena. Cancer is a complex disease process that requires overcoming several layers of protection. Thus, circadian modulation of this process may occur through any of these layers and will also be multi-faceted and complex. Several groups have used the power of mathematical modeling to improve our understanding not only of the cellular circadian clock but of these complex relationships as well ^9, 10, 82, 97, 98^. In addition to molecular connections between circadian clocks and pathways that influence transformation, circadian rhythms robustly influence the efficacy and toxicity of pharmacological compounds, including chemotherapy drugs ^99– 104^. Mathematical modeling of drug pharmacokinetics and pharmacodynamics is used by pharmaceutical companies in preclinical studies. Although the number of variables is a major obstacle to generating complete models, some groups have begun to incorporate circadian modulation of drug distribution and metabolism into so-called multi-scale pharmacokinetics models ^104^. Continued improvement of these models with the incorporation of new information emerging from the literature may lead to better pharmacological strategies.

Clocks may control many aspects related to all of the established and emerging hallmarks of cancer ^105^. Therefore, it is no wonder that results of *in vivo* studies have been variable depending on the method of clock disruption as well as the specific cancer model employed. Greater understanding of the interrelationship between circadian clocks, the cell cycle, and tumor formation and progression will enable improved lifestyle recommendations, occupational and public health policies, and pharmacological strategies ^100^ for the prevention and treatment of cancer.
